# A Report of Two Rare Cases of Buschke-Löwenstein Tumor

**DOI:** 10.7759/cureus.52700

**Published:** 2024-01-21

**Authors:** Tariq Bouhout, Abdelbassir Ramdani, Ayoub Kharkhach, Badr Serji

**Affiliations:** 1 Surgical Oncology, Mohammed VI University Hospital, Regional Oncology Center, Oujda, MAR

**Keywords:** bushke-löwenstein tumor, condyloma acuminata, local excision, giant condyloma acuminatum, human papillomavirus

## Abstract

The Buschke-Löwenstein tumor is a rare clinical entity. Its severity is related to the local invasion and the risk of recurrence and malignant transformation. It is caused by a viral infection due to the human papillomavirus. The transmission is primarily sexual and often affects the penile region. The perineal location is relatively rare. We report two rare cases of neglected Buschke-Löwenstein tumor due to the late diagnosis treated with large surgical resection. This study aimed to emphasize the contribution of clinical examination in the early diagnosis and the management of our patients.

## Introduction

Giant condyloma acuminatum or Buschke-Löwenstein tumor (BLT) is a large cauliflower-like budding tumor generally exceeding 10 cm in size presenting in the anogenital region caused by several types of human papillomavirus (HPV), the most common types involved are HPV 6, 11, 16, and 18 [1,2]. It is a sexually transmitted infection often affecting the penile region [3]. This slowly growing tumor is known for its local invasion and the high risk of recurrence and malignant transformation [4]. Here, we report two rare cases of BLT to emphasize the importance of a careful clinical examination for an early diagnosis and adequate management.

## Case presentation

Case 1

**Figure 1:**
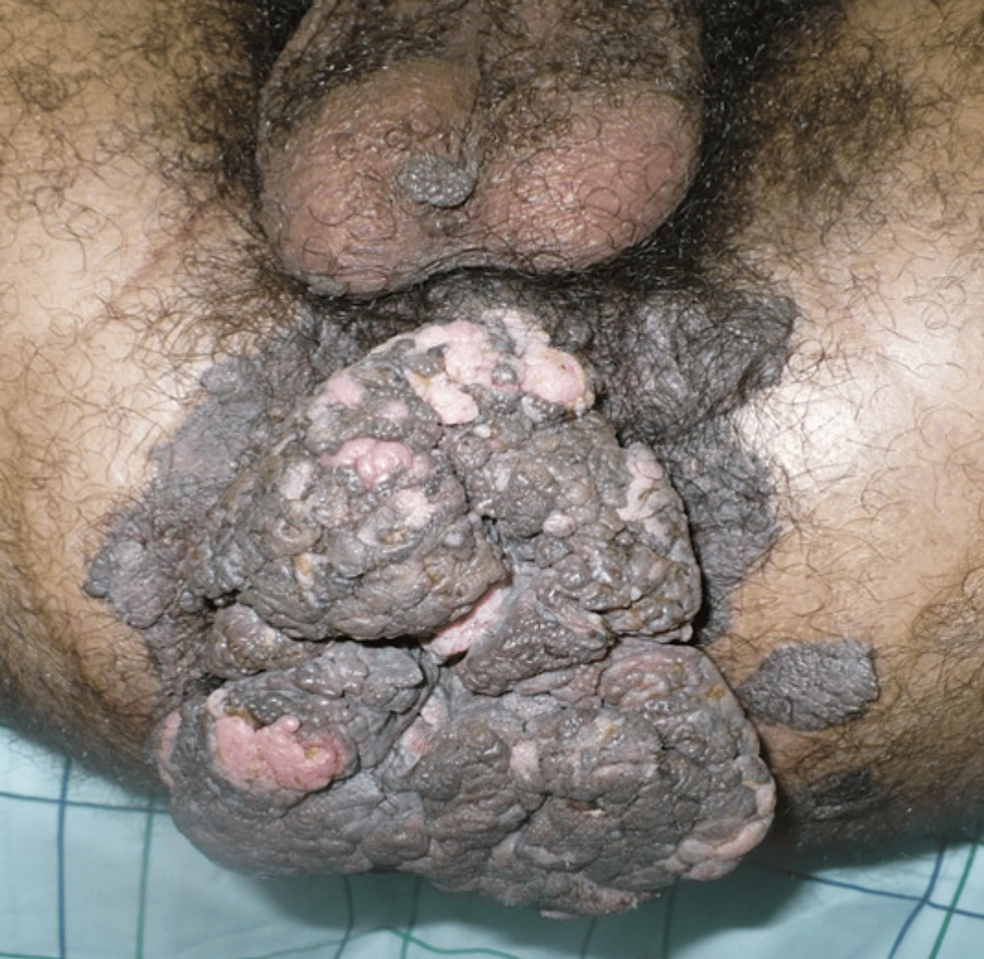
Cauliflower-like tumor covering the entire perineal region with multiple verrucous lesions in the left buttock.

A 34-year-old male patient, a chronic smoker with multiple sexual partners and a history of alcohol abuse presented with a perineal tumor evolving for 12 years. The clinical examination revealed a foul-smelling cauliflower-like tumor measuring 13×10 cm covering the entire perineal region as well as multiple verrucous lesions 2-3 cm in diameter in the left buttock (Figure 1).

**Figure 2:**
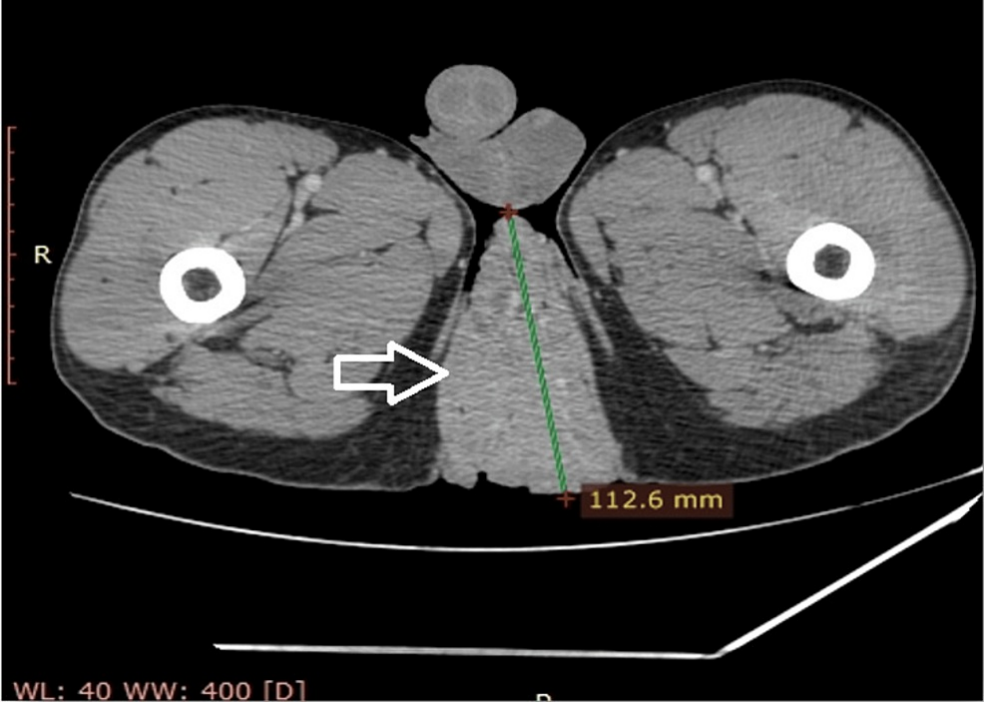
Axial CT scan showing the perineal tumor measuring 112.6 mm (arrow).

Bilateral painful inguinal lymphadenopathy was found on lymph node examination. Syphilis, hepatitis B, hepatitis C serologies, and human immunodeficiency virus (HIV) tests were all negative. Anoscopy revealed a congestive mucosa without any tumoral invasion. Abdominopelvic computed tomography (CT) revealed a perineal mass measuring 13×11×8 cm without an invasion of the posterior wall of the anal canal and the presence of bilateral inguinal lymphadenopathies (Figure 2).

**Figure 3:**
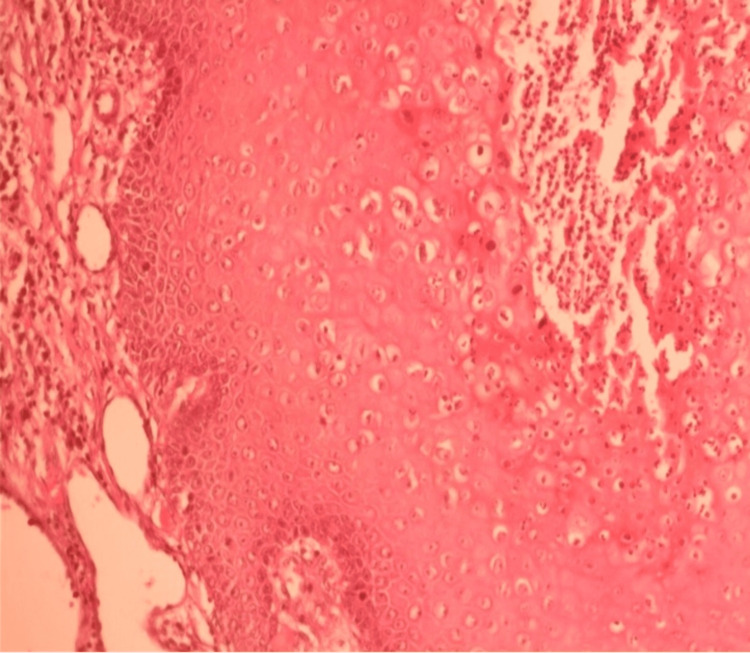
Pathological aspect showing squamous cells with epithelial hyperplasia, hyperacanthosis, hyperpapillomatosis, and koilocytes.

**Figure 4:**
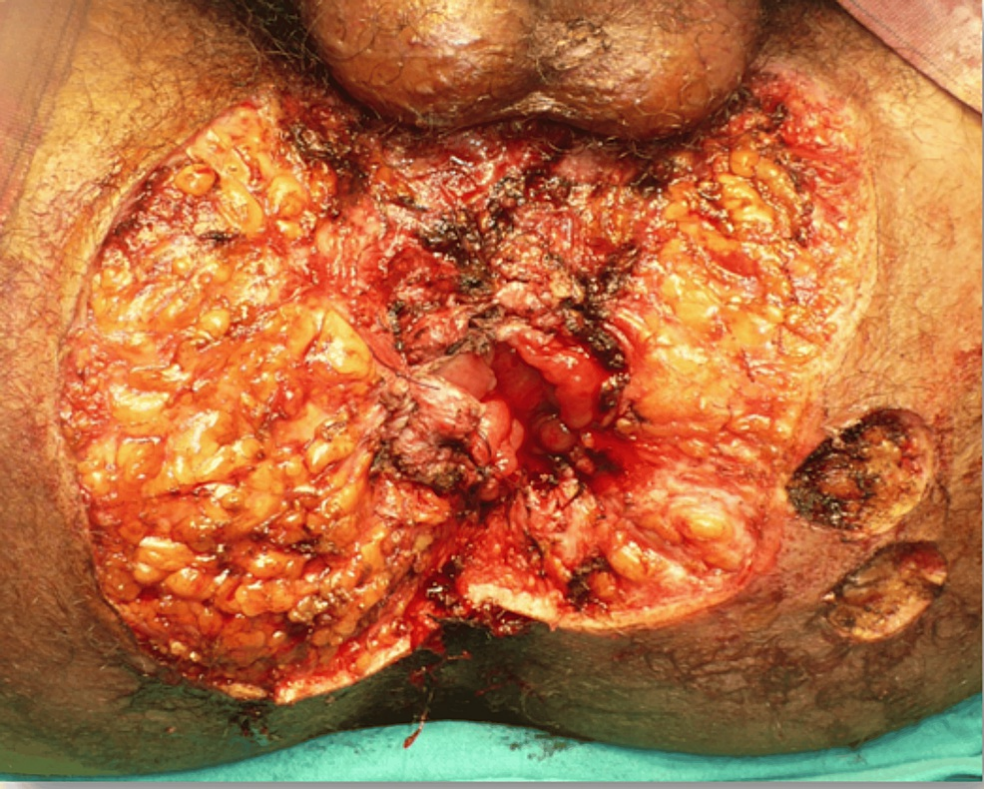
Immediate postoperative view after the complete resection of the tumor.

The pathological examination of a tumor biopsy revealed a squamous cell with epithelial hyperplasia, hyperacanthosis, hyperpapillomatosis, and koilocytes without signs of atypia or invasion of the dermis (Figure 3). Therapeutic management consisted of complete excision of the tumor with preservation of the anal canal without flap reconstruction (Figure 4). The pathological examination of the surgical specimen showed no signs of malignant transformation. The patient is seen regularly in consultation to assess wound healing.

Case 2

**Figure 5:**
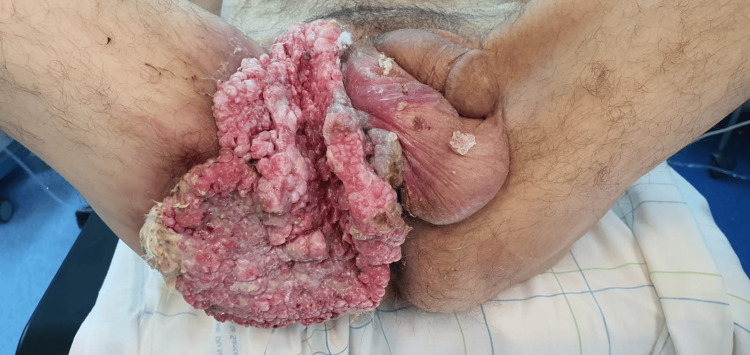
Visualization of the patient's tumor at the time of diagnosis.

A 66-year-old male patient with unknown sexual orientation presented with a perineal tumor that had evolved for 10 years and was treated inappropriately. The patient had no previous pathological history. The clinical examination revealed a cauliflower-like tumor involving the perineum, the right buttock and inguinal region, and part of the scrotum (Figure 5).

Syphilis, hepatitis B, hepatitis C serologies, and HIV tests were all negative. The abdominopelvic CT scan revealed a mass in the perineal region and the right buttock measuring 15×12×6 cm without invasion of the anal canal.

**Figure 6:**
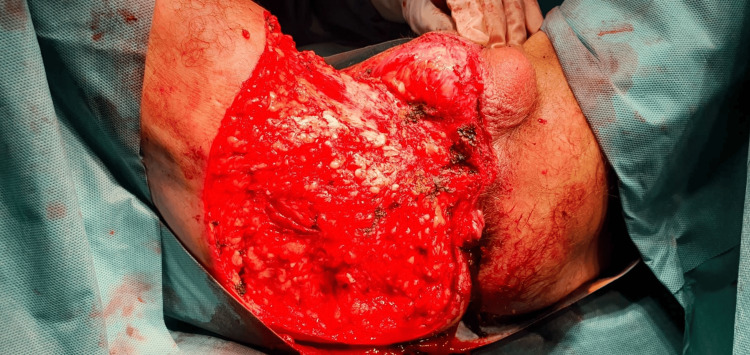
Immediate postoperative view after complete resection of the tumor.

The pathological examination of a tumor biopsy revealed a squamous cell with epithelial hyperplasia, hyperacanthosis, hyperpapillomatosis, and koilocytes without signs of atypia or invasion of the dermis. Therapeutic management consisted of the complete removal of the tumor without any flap coverage (Figure 6). The pathological examination of the surgical specimen showed no signs of malignant transformation. The patient was seen regularly in consultation to assess wound healing.

## Discussion

BLT is a sexually transmissible epithelial tumor most commonly found in the penile region [5]. Its incidence is estimated at 0.1% in the sexually active adult population [5]. A total of 81-94% of cases involve the penis, and only 10-17% involve the anoperineal region [6]. The perineal localization found in our patient is rare, with only 1.2 cases/year reported in the literature over the last millennium [4]. However, a new study has shown a slight recrudescence over the last 10 years, with 63 cases reported at a rate of 6.3 cases per year [2].

BLT is caused by HPV, with an incubation period of two to three months and up to 20 months. Transmission is essentially sexual [1,2]. Immunodeficiency, chronic irritation, ulcerative colitis, anal fistulas, and poor hygiene are all factors involved in the development of BLT [1,2].

A search for other associated sexually transmitted infections is necessary. A rigorous clinical examination and radiological examinations help to establish a precise lesion assessment. The pathological examination shows a papillomatosis, a hyperacanthosis with well-differentiated, regular epithelium and no cytonuclear abnormalities. In the superficial layers, there are koilocytes and the basement membrane is respected [7,8]. The clinical examination and the benign nature of the mass confirm the diagnosis [7].

The surgical treatment proposed to our patient at this stage remains the treatment of reference at the cost of significant loss of substance and a non-negligible risk of recurrence and carcinomatous transformation estimated at 30% after five years [2-4]. In perineal localizations, the resection with preservation and reconstruction of the sphincter is performed as often as possible, but more extensive operations involving abdominoperineal resection are sometimes performed [8]. Spontaneous regression of the tumor is exceptional, and recurrence may occur, especially after incomplete resection [8]. Early diagnosis is crucial for adequate management, it cannot be envisaged without careful clinical examination of our patients.

## Conclusions

BLT is a relatively rare clinical entity known for its local invasion, recurrence after treatment, and possible malignant transformation. A meticulous clinical examination is essential for adequate management. The two cases we reported presented a highly advanced tumor due to late diagnosis that could have been treated at an earlier stage with minimal resection.
